# Death of Mr. J. T. Harrison

**Published:** 1884-07

**Authors:** 


### DEATH OF MR. J. T. HARRISON.

It gives us pain to announce the death of this excellent gentleman, which occurred at Indian Springs a few days ago.

The JOURNAL assumed its present form and handsome dress under his directions.

We extend to the bereaved family our sincerest sympathies.

The following tribute to his memory is taken from the *Evening Journal* of this city, edited by a gentleman of high literary attainments, who was associated with the deceased at the Franklin for a number of years, and therefore knew him well.

" We regret to learn that Mr. John T. Harrison, the general superintendent of the Franklin Printing office, died at Indian Springs to-day of paralysis. Mr. Harrison has been in ill health for several years, and went to Indian Springs a month ago, hoping to be benefited by its healing waters.

" Mr. Harrison held a position of great responsibility in the Franklin Printing House, and was an excellent chief of his department. He was a man of many noble traits of character, loyal in his friendships, of quick impulses, generous to a fault, a devoted son, a kind brother and an honest hater of shams. During the late war he was a brave Confederate soldier, and had many friends in Georgia who will feel sincere regret over his untimely death. His remains will be taken to Milledgeville, to be buried in the old family burial grounds, by the side of his father, Hon. George W. Harrison, whom our old citizens will remember as formerly Secretary of State."

